# A characteristic image in Joubert syndrome: molar tooth sign

**DOI:** 10.11604/pamj.2015.21.69.7068

**Published:** 2015-05-28

**Authors:** Mouna Sghir, Wassia Kesomtini

**Affiliations:** 1 Unit of Physical Medicine and Rehabilitation, University Hospital Tahar Sfar, Mahdia, Tunisia

**Keywords:** Joubert syndrome, molar tooth, aplasia

## Image in medicine

Joubert syndrome is a relatively rare autosomal recessive congenital disorder; it is characterized by cerebellar vermis hypoplasia or aplasia. Characteristic clinical symptoms and signs include motor and respiratory abnormalities. It is currently included in the malformation spectrum of cerebello-oculo-renal syndromes (CORS). An image known as a “molar tooth sign” is typically observed in cerebral magnetic resonance imaging (MRI) and is characterised by a deep posterior interpeduncular fossa, thickened and elongated superior cerebellar peduncles, as well as hypoplasia or agenesis of the cerebellar vermis. We report the case of a 4-year-old male, referred to our rehabilitation unity with a history of hypotonia and delayed psychomotor development. Physical examination found macrocephaly, frontal bossing and triangular upper lip and arched palate. Ocular examination revealed a bilateral divergent squint and inability to track objects with eyes. All aspects of his development were delayed. He had a generalized hypotonia but deep tendon reflexes were normal. There were important negative signs including: Regular breathing pattern, no organomegaly and no polydactyly or syndactyly. With these findings, a brain MRI was requested, which showed the classic “molar tooth sign” which led to the clinical diagnosis of Joubert syndrome. In complementary studies, the audiogram revealed a bilateral sensorineural hearing loss, the ophthalmology assessment and laboratory studies were normal. We have prescribed a stander and hearing aid. A rehabilitation program was started consisting of: joint mobilization, muscle strengthening, occupational and speech therapy.

**Figure 1:**
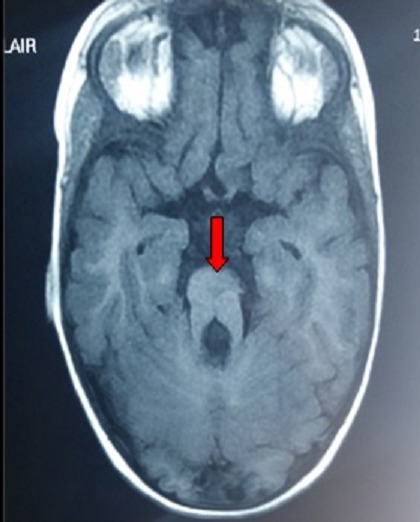
Cerebral MRI showing agenesis of cerebellar vermis causing the “molar tooth sign”

